# Mesonephric-like adenocarcinoma as an unexpected histological result after fertility saving procedure for presumed adenomyosis: a case report

**DOI:** 10.3389/fonc.2026.1828586

**Published:** 2026-06-05

**Authors:** Vojtěch Lukavec, Filip Frühauf, Zdenka Lisa, Jan Galko, Pavel Dundr, Michal Mara

**Affiliations:** 1 st Center for Minimally Invasive Gynecologic Surgery, Department of Gynecology, Obstetrics and Neonatology, General University Hospital in Prague and the 1 Medical Faculty of the Charles University, Prague, Czechia; 2 st Institute of Pathology, General University Hospital in Prague and the 1 Medical Faculty of the Charles University, Prague, Czechia

**Keywords:** adenomyosis, endometriosis, fertility saving surgery, mesonephric-like adenocarcinoma, unexpected histology

## Abstract

**Introduction:**

We report an unexpected case of mesonephric-like adenocarcinoma initially treated as a diffuse adenomyosis in a patient presenting with primary infertility. The aim of this report is to raise clinical awareness about the risk of a diagnostic pitfall where an unexpected malignancy can mimic diffuse adenomyosis. The patient’s main concerns and important clinical findings: A 41-year-old patient referred to a tertiary minimally invasive gynecological center in June 2023 for primary infertility, heavy menstrual bleeding, and a 5 x 6 cm myometrial mass diagnosed as diffuse adenomyosis on ultrasound scan.

**The primary diagnoses, interventions, and outcomes:**

An open cytoreductive resection of adenomyosis with uterine cavity reconstruction was planned due to worsening abnormal uterine bleeding and two failed *in vitro* fertilization attempts. An unexpected Mesonephric-like adenocarcinoma with only small foci of healthy tissue was identified on histopathological examination, and immunohistochemical staining. Consequently, the patient had a total abdominal hysterectomy with bilateral salpingo-oophorectomy and systematic pelvic lymphadenectomy. This was followed by six cycles of paclitaxel and carboplatin plus pembrolizumab-based immunotherapy.

**Conclusions:**

This case highlights the importance of thorough evaluation of all atypical myometrial lesions, including adenomyosis, where pre-conception surgical excision and histopathological evaluation are often deferred in most patients.

## 1 Introduction

Despite being one of the frequent co-factors in infertility, adenomyosis continues to present a significant management challenge in reproductive medicine. Debulking cytoreductive surgery for diffuse adenomyosis is a technically demanding and controversial procedure associated with high risk of complications, including uterine rupture in subsequent pregnancies (1, 2). Therefore, it is often offered as a last resort for symptomatic patients who are resistant to hormonal treatment and assisted reproductive techniques.

It is considered good clinical practice to base the diagnosis of adenomyosis on a thorough evaluation of patients’ symptoms, reproductive history, and ultrasonographic imaging using the revised MUSA (Morphological Uterus Sonography Assessment) criteria, which serve as the gold standard for describing myometrial lesions (3, 4). However, despite meticulous adherence to the recommended clinical protocol, there are case reports of malignant transformations or unexpected malignancies that mimic adenomyosis on ultrasound or MRI (5, 6).

Malignant transformation of adenomyotic lesions is considered a very rare complication, typically affecting elderly patients rather than younger women seeking fertility-sparing surgery to improve their reproductive outcomes (7). Based on their histopathological origin, adenomyosis-associated malignancies are classified into epithelial (typically endometrioid or clear cell carcinoma), and mesenchymal malignancies (e.g. endometrial stromal sarcoma).

Mesonephric-like adenocarcinoma (MLA), categorized in the 2020 WHO Classification of Female Genital Tumors, is a recently described epithelial subtype (8). MLA lacks clear mesonephric remnants and consists solely of cells of Müllerian origin or secondarily transformed cells. The median age at diagnosis of this pathology is 61 years, with a range of 36 to 76 years. It shares many characteristics with the more common mesonephric adenocarcinoma, which predominantly arises from the uterine cervix (9–11).

Mesonephric adenocarcinoma is generally believed to originate from remnants of the embryonic mesonephric (Wolffian) duct. Islets of this residual tissue are typically found in the paraovarian region and deep within the cervical stroma, which correlates with the sites where this tumor is most frequently described in case series (12, 13). Although mesonephric-like adenocarcinoma shares histological properties with this group of tumors, it is found in tissues that are not embryologically linked to the Wolffian system. Moreover, despite the significant molecular similarity in the activation of KRAS mutations, MLA lacks PTEN mutations found in mesonephric adenocarcinomas originating from the mesonephric duct (14).

It has been suggested that MLA of the uterine body can arise in patients previously diagnosed with adenofibromas and adenomyosis (15). MLA of the uterine body tends to exhibit a large tumor size, diffuse and endophytic growth into the myometrium, strong contrast enhancement on Fs-Gd GRE T1WI, and restricted diffusion on DWI. The frequency of advanced-stage disease (FIGO stages III-IV) is significantly higher in MLA (63.2%) compared to endometrioid endometrial cancer (11). Additionally, MLA of the uterine body is prone to early recurrence and distant metastases, most commonly to the lungs. Abnormal uterine bleeding, including menorrhagia, is a frequent though nonspecific symptom of this pathology (16).

In this report, we describe a case of unexpected MLA diagnosed on histopathological examination of specimens removed during an open cytoreductive procedure for suspected adenomyosis.

## 2 Case description and diagnostic assessment

A 41-year-old patient was referred to our center in June 2023 with a 12-month history of primary infertility, dysmenorrhea, menorrhagia, and a 5 x 6 cm myometrial mass suspected to be an intramural fibroid. She was taking thyroxine for hypothyroidism and had mild anemia, for which she started iron supplementation.

A combined transvaginal and transabdominal ultrasound performed in August 2023 revealed an atypical myometrial lesion measuring 60 x 55 x 47 millimeters, with non-uniform myometrial echogenicity, with hyperechogenic islands, anechoic cysts, and fan-shape shadowing. The margins and junctional zone of the lesion were ill-defined. There was asymmetry between the uterine walls, with the anterior wall more than three times thicker than the posterior. The lesion was predominantly localized in the anterior wall and along the left uterine margin and was described as diffuse adenomyosis. There was no evidence of other endometriotic lesions or other pelvic pathology.

In view of the diagnosis and absence of other pathologies, the patient was counseled about and consented to be enrolled into the REAdME study, a prospective, non-randomized study of molecular markers of endometrial receptivity and fertility outcomes in patients with uterine fibroids, adenomyosis, and endometriosis (ClinicalTrials.gov ID: NCT06991595) Within the study, the patient opted for the non-surgical management option and was scheduled for an *in-vitro* fertilization program.

Following her first unsuccessful embryo transfer, the patient underwent a diagnostic hysteroscopy, which demonstrated a symmetrical uterine cavity with no visible signs of the adenomyotic lesions or any other pathology. An endometrial biopsy revealed a normal secretory endometrium without any significant abnormalities. The patient then had a failed cryo-embryo transfer.

In view of the two failed IVF attempts, and worsening abnormal uterine bleeding symptoms, she was offered an open cytoreductive resection of adenomyosis to increase her chances of subsequent IVF success and agreed to proceed.

**Figure 1:**
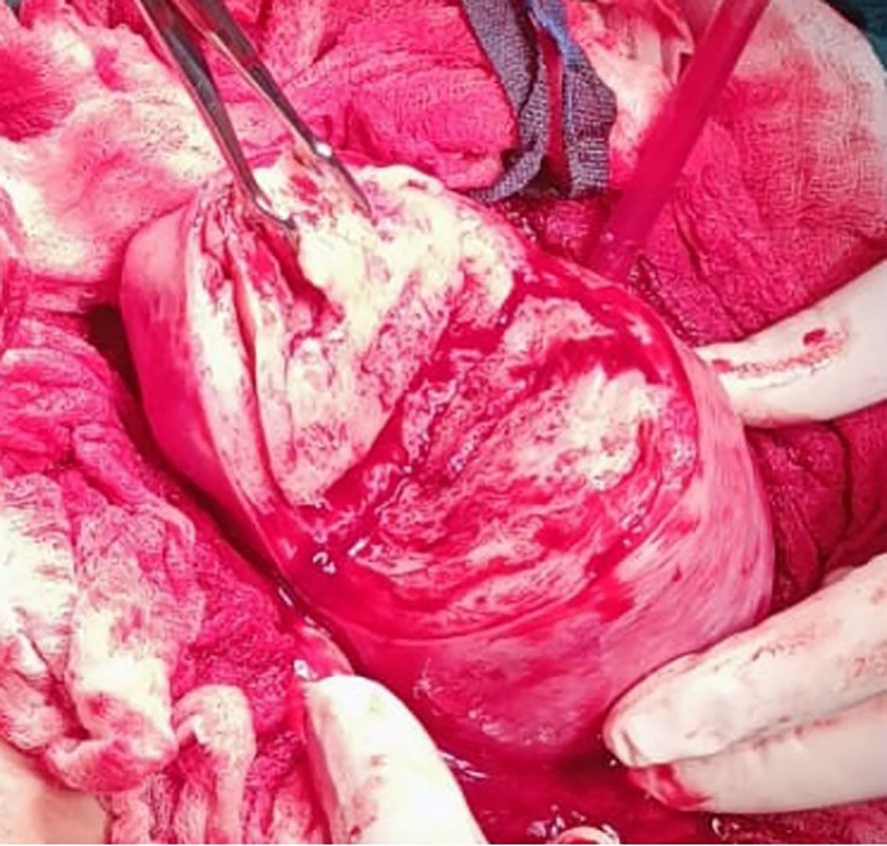
Intraoperative image demonstrating the site of uterine incision over the lesion site breaching the uterine cavity.

Surgery was performed in June 2024. An initial diagnostic laparoscopy was conducted to assess the pelvis, followed by a planned laparotomy, which was considered more suitable given the size of the lesion and the need for uterine cavity reconstruction. The laparotomy was performed through a Pfannenstiel suprapubic incision. Following parametrial dissection and mobilization of both ureters, a uterine artery tourniquet was applied, and the ovarian ligaments were clamped to minimize bleeding. This was followed by a uterine incision over the lesion site till the uterine cavity (**Figure 1**). After removal of all palpable parts of the firm pathological tissue, the uterus was reconstructed using Osada’s modified triple-flap technique (2). The lesion was confined to the myometrium without any involvement of the serosa or the uterine cavity. Apart from patterns of firm fibrotic myometrial remodeling typical of diffuse adenomyosis, there were no other unexpected intraoperative findings. The operative time and estimated blood loss were 85 minutes and 800 mL respectively. The patient had an uneventful postoperative course and was discharged on the third postoperative day, with a plan for IVF in 6 months.

**Figure 2:**
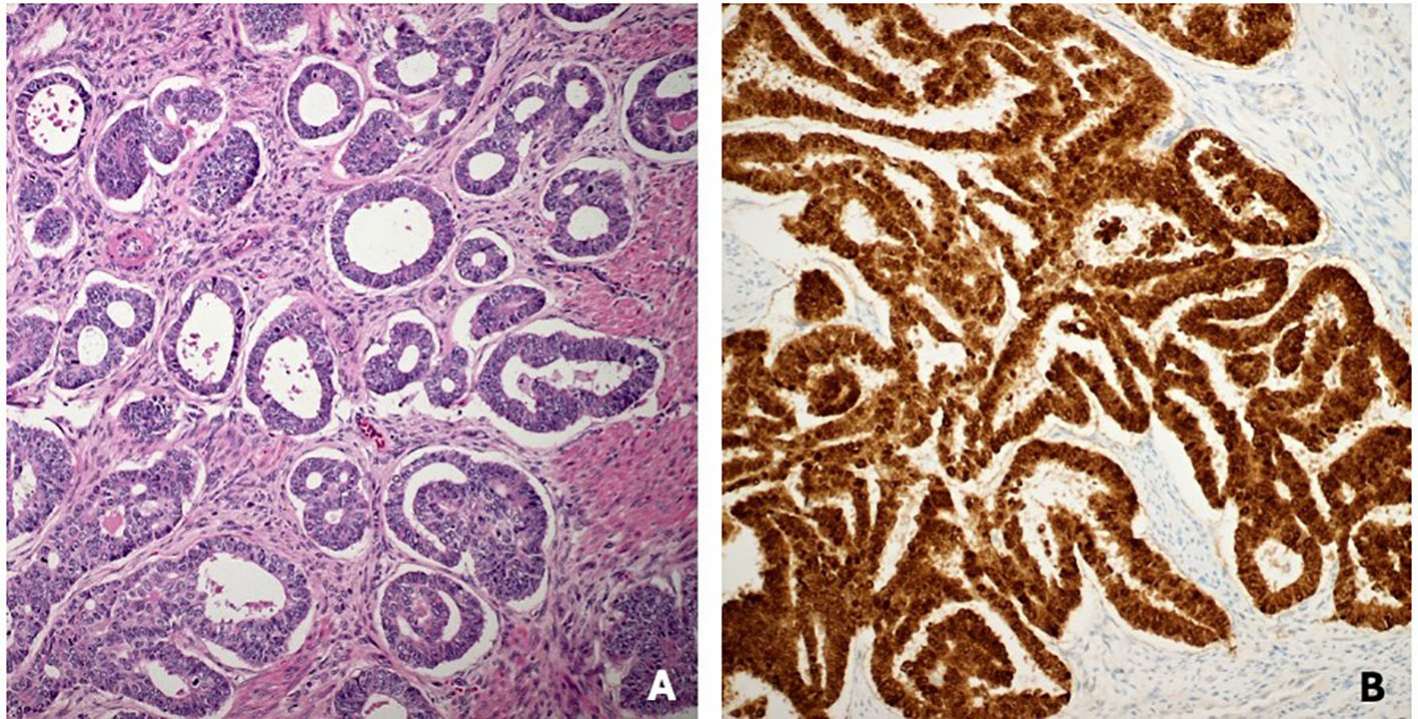
Histopathological images of the tumor (H&E stain [2A] and PAX8 immunohistochemical stain [2B]) demonstrating the microanatomy and structure of the resected tumor. Both images show diffuse infiltration of healthy spindle-shaped smooth muscle cells of the myometrium. MLA may form a variety of microanatomical structures, including villoglandular, papillary (sometimes resembling serous borderline ovarian tumor or papillary thyroid carcinoma), tubular, and cribriform patterns. In section 2A, predominantly tubular and cribriform adenocarcinoma structures are present, formed by cuboidal to columnar cells with irregularly distributed polygonal cells. The cell nuclei are mostly uniform and round, with finely dispersed chromatin and inconspicuous nucleoli. The cytoplasm of the MLA cells is moderate in volume and moderately to strongly eosinophilic. MLA tissue may also contain areas of necrosis and hemorrhage; however, these features are not prominent in the examined sections. Diffuse PAX8 positivity in section 2B is typical of MLA and other tumors of Müllerian origin. PAX8 is a nuclear marker expressed in the epithelium of the Müllerian tract and prominently stains tumor cell nuclei dark brown. In our case, the tumor also demonstrated positivity for TTF-1 and low-level positivity for GATA3, with these two markers typically exhibiting an inverse staining pattern. Original magnification ×200.

**Figure 3:**
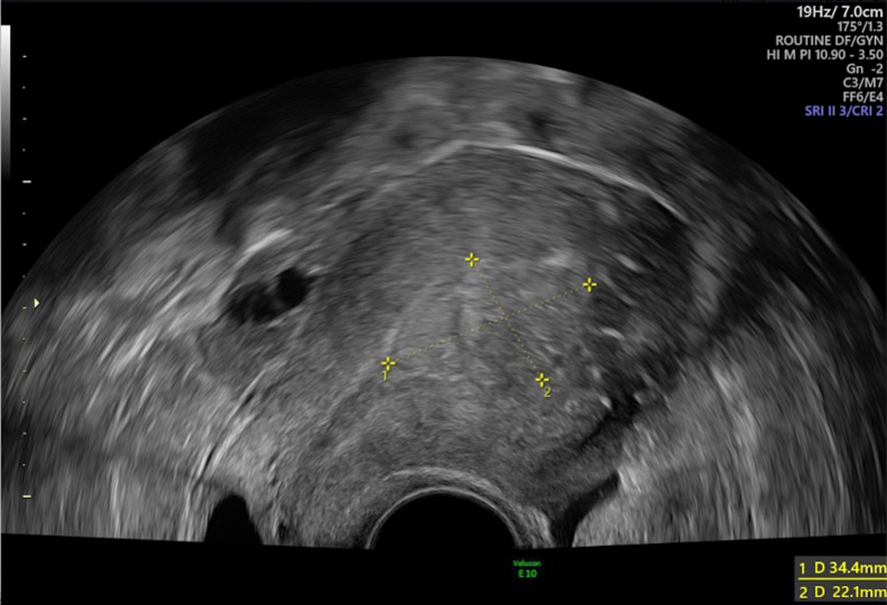
Image captured at the time of ultrasonographic staging of the tumor, displaying uterine cavity, residual lesion, and visible suture in the uterine fundus after previous surgery.

Unexpectedly, histological analysis and subsequent immunohistochemical staining revealed an extensive presence of mesonephric-like adenocarcinoma cells, with only a minor presence of endometrial cells in the specimen (**Figure 2**). The patient underwent a preoperative diagnostic work-up for endometrial cancer, including ultrasonography for local staging and a chest X-ray (**Figure 3**). Ultrasound classified the endometrial cancer as FIGO stage II, with deep myometrial invasion and cervical stromal involvement. Following discussion at our Gyne-oncology multidisciplinary team meeting, the patient was scheduled for a total abdominal hysterectomy with bilateral salpingo-oophorectomy and sentinel lymph node biopsy. Sentinel lymph node mapping was unsuccessful, and a systematic pelvic lymphadenectomy was therefore performed. Apart from two suspicious pelvic lymph nodes, no other macroscopic evidence of peritoneal disease was observed. Subsequent histopathological assessment showed that there was an 80 x 30 x 40 mm MLA lesion with deep myometrial invasion, cervical stromal infiltration, and a 10 mm metastasis in the right ovary. There was also a 1 mm implant on the left ovary. Two of the 16 removed lymph nodes (right common iliac and left external iliac) were infiltrated.

Due to this unexpected histological over-staging, a postoperative CT scan was performed, which confirmed extensive peritoneal involvement with diffuse carcinomatosis on the diaphragm, liver, sigmoid colon, ileum, an enlarged paraaortic lymph node (43 mm in diameter), and suspicious mediastinal lymph nodes. Based on these findings, the stage was determined to be pT3a, pN1 (16/2), FIGO IIIC1. Using molecular classification, endometrial cancer was categorized as no special molecular profile [NSMP] (i.e. microsatellite stable with wild type expression of p53 and no pathogenic variant of POLE). DNA and RNA NGS was performed on platform NextSeq 2000 using the sequence capture NGS method with TMB (tumor mutational burden) cutoff being 10 mutations per megabase. Selected somatic variants of 360 genes were investigated, also including CTNNB1 (13% mutation frequency) and KRAS (61% mutation frequency). Mutations detected were CTNNB1; NP_001895.1:p.Ser37Phe, p.(Ser37Phe) and KRAS; NM_033360.4:c.35G>A, p.(Gly12Asp).

**Figure 4:**
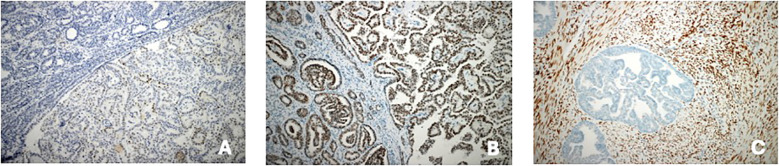
Section **(A)** - GATA3: Nuclear transcription factor, displaying mild positivity in fraction of nuclei of the tumor cells in the specimen (colored into dark brown). Staining often complementary with the SOX10 stain (not performed in this clinical case) highly sensitive for mesonephric lesions. This marker is usually not recommended for standalone use- it tends to be positive in urothelial carcinomas, breast carcinomas and many other clinical units including squamous cell lesions. Section **(B)** - TTF1: Thyroid transcription factor 1 shows very high nuclear stain positivity in pseudo-glandular structures of the tumor, marking them with dark brown/black color while healthy myometrial smooth muscle cells being negative. Marker is expressed in high percentage of adenocarcinomas and usually forms inverse cell stain patterns compared to GATA3. Section **(C)** – ER: Estrogen receptors being negative in tumor cells typically support the diagnosis of MLA- same as the negativity of PR (progesterone receptor). Healthy myometrial cells are being stained with brown color, while central dominant tumor structure remains negative.

There was no expression of estrogen or progesterone receptors, which is considered typical for MLA. Tumor cells tested positive for PAX8, TTF1, with minor positivity for GATA3 (**Figure 4**).

In December 2024, the patient completed 6 cycles of carboplatin AUC 5 and paclitaxel 175 mg/m2 combined chemotherapy without any severe toxicity. As the tumor mutation burden (TMB) was high (15 mut/Mb), pembrolizumab, a checkpoint inhibitor used in cancer immunotherapy, was added concurrently with the chemotherapy and continued as maintenance therapy. CT scans performed during and after the completion of chemotherapy confirmed a partial response in all regions, including both the carcinomatosis and lymph nodes. Pembrolizumab was administered until disease regression was documented on CT scan in May 2025. Based on CT scan follow-up in May 2025, the only sites of treatment failure were the paraaortic lymph nodes. A secondary cytoreductive surgery was performed in June 2025 to remove bulky lymph nodes in the paraaortic region located inferior to the left renal vein. Intraoperatively, no peritoneal spread was observed, and optimal nodal debulking with no residual disease was achieved. After this surgical procedure, the patient started second-line chemotherapy including carboplatin (CBDCA) and doxorubicin. The patient received the sixth CBDCA cycle in November 2025. Treatment was complicated by deep venous thrombosis of the left leg and bilateral pulmonary embolisms, which were managed medically. The patient also reported newly developed tinnitus, likely a side effect of cisplatin therapy. Although the disease appears controlled at the time of this report, the prognosis remains uncertain, particularly considering treatment-related side effects. A timeline of clinical events is presented in **Table 1**.

**Table 1:** Timeline of clinical events.

Date/Timeframe	Event	Details
June 2023	Initial referral	41-year-old patient referred with a 12-month history of primary infertility, dysmenorrhea, menorrhagia, and 5x6 cm suspected intramural fibroid. On thyroxine for hypothyroidism. Mild anemia treated with iron supplementation.
August 2023	Pelvic ultrasound assessment	Combined transvaginal and transabdominal ultrasound showed 60x55x47 mm atypical myometrial lesion with diffuse adenomyosis features. No other endometriotic or pelvic pathology.
November 2023 – February 2024	Enrollment in REAdME study (ClinicalTrials.gov ID: NCT06991595).	Opted for non-surgical management and IVF program. Two failed IVF cycles and normal diagnostic hysteroscopy in between.
June 2024	Open cytoreductive surgery for adenomyosis	Diagnostic laparoscopy followed by laparotomy via Pfannenstiel incision. Lesion resected and uterus reconstructed. Histology revealed extensive mesonephric-like adenocarcinoma (MLA) with minor benign adenomyosis.
Mid–Late 2024	Preoperative cancer staging and definitive surgery	Ultrasound and chest X-ray performed. Classified as FIGO stage II (deep myometrial invasion and cervical stromal involvement). Total abdominal hysterectomy, bilateral salpingo-oophorectomy and pelvic lymphadenectomy.
Late 2024	Postoperative CT & final staging	Final stage: pT3a, pN1 (2/16) FIGO IIIC1. Molecular subtype: NSMP. ER/PR negative.
December 2024	Completion of first-line chemotherapy	6 cycles carboplatin (AUC 5) + paclitaxel (175 mg/m2). Pembrolizumab added due to high TMB (15 mut/Mb). Partial radiologic response.
May 2025	Follow-up CT evaluation	Partial response maintained. Persistent paraaortic lymph node disease identified.
June 2025	Secondary cytoreductive surgery	Removal of bulky paraaortic lymph nodes under left renal vein. No peritoneal spread. Complete nodal debulking achieved.
June–November 2025	Second-line chemotherapy	Carboplatin (CBDCA) + doxorubicin. Sixth cycle completed November 2025.
During 2025 treatment	Treatment complications	Deep venous thrombosis (left leg), bilateral pulmonary embolisms (medically managed), and tinnitus (likely platinum-related).
At time of case report	Current status	Disease under control; prognosis uncertain due to aggressive biology and treatment-related complications.

## 3 Discussion

Despite advances in imaging modalities, accurate evaluation of certain uterine lesions remains a diagnostic challenge. The incidence of benign uterine myometrial lesions, mainly uterine fibroids, but also adenomyosis, is high among women of reproductive age compared to the incidence of malignancies in this group, making the clinical suspicion and diagnosis of cancerous and pre-cancerous uterine pathologies less likely.

The diagnostic and management protocols for rare uterine malignancies were recently described in the updated European Society of Gynecological Oncology (ESGO), the European Reference Network on Rare Adult Solid Cancers (EURACAN), and the Gynecologic Cancer InterGroup (GCIG) guidelines. Although developed to be primarily used for uterine sarcomas, they are also applicable to other myometrial malignancies (17). An approach similar to the preoperative biopsy of atypical myomas is recommended for assessing adenomyotic lesions. However, the physical characteristics of these lesions may pose challenges for the technique of tru-cut biopsies (17). Transvaginal biopsies of adenomyotic lesions have shown promising diagnostic accuracy, with a concordance rate of 92.6% between biopsy results and postoperative histology (18). Additionally, ultrasonography enables the assessment of other diagnostic criteria, including the size of the dominant locus of the lesion and its detailed ultrasound characteristics.

The five-year survival of MLA is currently estimated at 71% and 72%, which is among the lowest of endometrial carcinoma subtypes. This places it between the average survival rate for endometrial carcinoma (approximately 82%) and the dismal prognosis for metastatic uterine sarcoma, with a five-year survival rate of 10% to 15% (19). Its early tendency to form micrometastases complicates prognostic evaluation based on initial staging and renders fertility-sparing surgical approaches nonviable. Furthermore, analyses of patients diagnosed with this cancer subtype revealed a heightened tendency for recurrences (20). It has also been reported that only one-third of MLAs were correctly diagnosed on initial biopsy, highlighting the importance of an experienced pathologist in the diagnostic process (21).

The occurrence of these cancer subtypes raises important questions regarding the surgical treatment of large adenomyotic lesions, potentially supporting earlier surgical intervention in women planning to conceive, especially those over the age of 40. MLA and other rare cancer variants are infrequent, yet because of their clinical significance, they should be included in the differential diagnosis of symptomatic patients with myometrial lesions. Further research is needed in this area, and additional data are necessary to establish appropriate management algorithms, particularly regarding risk factors that contribute to the development of these malignancies in younger women.

Endometriosis and ectopic endometrium are conditions that can contribute to the development of various malignancies, including endometrioid carcinoma and clear cell carcinoma. Emerging evidence also suggests their involvement in MLA, especially its extrauterine forms, such as ovarian or peritoneal MLA (22, 23). Although there is currently no strong evidence establishing direct pathophysiological steps between adenomyosis and the development of MLA, recent literature indicates a high co-occurrence of adenomyotic lesions and shared KRAS mutations in both tumors and ectopic endometrial tissue (24). Advances in understanding this malignancy may facilitate the development of more targeted oncological treatments, whereas surgical management strategies remain largely unchanged. While multidisciplinary teams (MDTs) continue to play a key role in the management of certain pathologies, the role of molecular tumor boards (MTBs) is increasingly important, especially in diseases requiring diverse therapeutic strategies based on molecular subtyping, such as MLA.

In this case, based on the histopathological material processed, we cannot draw a definitive conclusion that malignancy coexisted with a diffuse distribution of benign adenomyosis, due to the predominance of tumor cells in the samples. The sparse distribution of endometrial cells observed in the histological images may represent either residual cells from a pre-existing adenomyotic lesion or displaced eutopic endometrium secondary to intraoperative uterine cavity breach.

A key limitation of our conclusions is the small number of cases reported in the literature for these malignancies, particularly in women initially managed with fertility-sparing procedures. Nonetheless, our case report underscores the possibility of a potentially aggressive disease morphologically mimicking a typically benign adenomyotic lesion that would not ordinarily warrant early surgical intervention or biopsy. Although rare, this condition should be considered in the differential diagnosis by specialists in reproductive medicine.

## 4 Patient perspective (translated from Czech)

The diagnosis came as a complete surprise to me, as I had not expected a serious underlying condition. The news was difficult to process, particularly given the absence of prior warning signs that I would have recognized as significant. After detailed discussions with the medical team, I agreed to the proposed treatment plan. During the course of my illness, I experienced complications related to deep vein thrombosis and pulmonary embolism, which were alarming and required additional medical management. These events added to my physical discomfort and emotional stress, but I felt they were addressed promptly and thoroughly by the treating physicians.

At one point, disease progression was identified despite ongoing therapy, which was discouraging. However, an alternative treatment strategy was initiated, and I appreciated being informed about the rationale behind this change in management. The subsequent therapy was associated with side effects, including tinnitus, which affected my daily activities and quality of life. Despite these challenges, I remained engaged in the treatment process and valued the clear communication and coordinated care provided by the multidisciplinary team. Sharing my experience highlights both the medical and personal complexities of cancer treatment from a patient’s perspective.

## Data Availability

The raw data supporting the conclusions of this article will be made available by the authors, without undue reservation.
